# Genome-wide analysis of WRKY gene family and the dynamic responses of key WRKY genes involved in cadmium stress in *Brassica juncea*

**DOI:** 10.3389/fpls.2024.1465905

**Published:** 2024-10-10

**Authors:** Shaocui Li, Qingqing Ji, Xia An, Changli Chen, Xiahong Luo, Tingting Liu, Lina Zou

**Affiliations:** Zhejiang Xiaoshan Institute of Cotton & Bast Fiber Crops, Zhejiang Institute of Landscape Plants and Flowers, Zhejiang Academy of Agricultural Sciences, Hangzhou, China

**Keywords:** cadmium, *Brassica juncea*, WRKY gene family, gene expression, cadmium stress

## Abstract

The WRKY transcription factors comprise one of the most extensive gene families and serve as pivotal regulators of plant responses to heavy metal stress. They contribute significantly to maintaining plant growth and development by enhancing plant tolerance. However, research on the role of WRKY genes in response to cadmium (Cd) stress in mustard is minimal. In this study, we conducted a genome-wide analysis of the mustard WRKY gene family using bioinformatics. The results revealed that 291 WRKY putative genes (*BjuWRKY*s) were identified in the mustard genome. These genes were categorized into seven subgroups (I, IIa-e and III) through phylogenetic analysis, with differences in motif composition between each subgroup. Homology analysis indicated that 31.62% of the genes originated from tandem duplication events. Promoter analysis revealed an abundance of abiotic stress-related elements and hormone-related elements within the *BjuWRKY* genes. Transcriptome analysis demonstrated that most *BjuWRKY* genes exhibited differential expression patterns at different Cd treatment stages in mustard. Furthermore, 10 *BjuWRKY* genes were confirmed to respond to Cd stress through the construction of a BjuWRKY protein interaction network, prediction of hub genes, and real-time fluorescence quantitative PCR analysis, indicating their potential involvement in Cd stress. Our findings provide a comprehensive insight into the WRKY gene family in mustard and establish a foundation for further studies of the functional roles of *BjuWRKY* genes in Cd stress response.

## 1 Introduction

Heavy metals constitute a pervasive class of pollutants that have garnered global attention (Kamal et al., 2023). Among them, cadmium (Cd) stands out as one of the most common harmful heavy metals and is extremely toxic. As agriculture and industry continue to expand and development, Cd spreads rapidly in ecosystems through processes such as mining, industrial emissions and pesticide use (Li et al., 2021). Despite being non-essential elements without a known biological function in living organisms, they can still be absorbed and utilized by plants through their natural ion uptake channels (Liu et al., 2019). In addition, Cd has high hydrophilicity and mobility and is easily taken up by plants from the soil (Wei et al., 2024). Excessive levels of Cd prevent plant uptake and transfer of nutrients and water, resulting in reduced seedling growth rate and root activity (Yang et al., 2022). Cadmium that enters the plant through soil contamination can accumulate within the plant tissues and ultimately end up in the human body along the food chain (Li S. et al., 2023; Liu et al., 2017).

Transcription factors contain numerous phosphorylation sites, which serve as key regulators of cellular responses to heavy metal stress by controlling the expression of downstream genes (Li et al., 2022). Furthermore, they are also central components of the regulatory network for heavy metal detoxification and tolerance. Many transcription factors intimately related to heavy metal detoxification and resistance pathway have been identified in plants. Among them, transcription factors such as basic leucine zipper (bZIP) (Han et al., 2021), basic helix-loop-helix (bHLH) (Yao et al., 2018), myeloblastosis (MYB) (Agarwal et al., 2020), and ethylene response transcription factor (ERF) (Lin et al., 2017) have been shown to play important roles in the regulation of heavy metal stress in plants.

The WRKY family, comprising one of the largest families of transcription factors in plants and plays a pivotal role in various biological processes, including growth, development and stress response, particularly in the context of heavy metal toxicity and resistance (Hu et al., 2021; Li et al., 2022; Wani et al., 2021). WRKY protein belongs to plant specific transcription factors, named for its N-terminus containing a special distinctive seven-amino-acid conserved sequence WRKYGOK (Xiong et al., 2024). Additionally, the WRKY transcription factor possesses a highly conserved WRKY structural domain, which comprises about 60 amino acids at the N-terminus, and a novel zinc finger motif C2H2 (C–X4–5–C–X22–23–H–X–H) or C2HC (C–X7–C–X23–H–X– C) at their C-terminus (Xie et al., 2018). WRKY proteins induce or repress the expression of their downstream genes by specifically binding to the W-box [TGACC (A/T)] at the promoter site (Qin et al., 2022; Zentgraf et al., 2010). Based on the number of WRKY structural domains and the type of zinc finger motif, WRKY proteins can be categorized into three classes: proteins containing two WRKY structural domains belong to class I, while proteins with only one WRKY structural domain belong to classes II and III. Members of classes I and II have C2H2 zinc finger-like motifs, whereas class III WRKY proteins contain C2HC zinc finger-like motifs (Eulgem et al., 2000).

Numerous evidences indicate that WRKY transcription factors play crucial roles in plant growth, development, and hormone regulation. A previous comparative transcriptomic study on cotton somatic embryogenesis revealed that 4.8% of the gene coding for WRKY transcription factors were detected during this process. Further research has demonstrated that *GhWRKY15* not only participates in the regulation of rhizome development but also enhances resistance to viral and fungal infections in tobacco when overexpressed (Sun et al., 2018; Yu et al., 2012). In another study, Xiong et al. (2024) identified 89 *Pisum sativum* WRKY genes and found that hormone response elements (ABA\ MeJA\GA SA\IAA) were present in several *Pisum sativum* WRKY genes. Expression patterns varied across different tissues and fruit developmental stages, as well as in response to hormone treatments. It was speculated that the *PsWRKYs* regulate *Pisum sativum* growth and development through hormone-mediated signaling pathways (Xiong et al., 2024). Additionally, overexpression of *TaWRKY75-A* and *ZmWRKY79* in *Arabidopsis thaliana* has been shown to improves plant survival under drought stress by regulating ABA biosynthesis (Gulzar et al., 2021; Ye et al., 2021).

In addition, WRKY transcription factors have been shown to play an equally important role in abiotic stress response. Qin et al. (2022) identified 113 WRKY genes from the apple genome GDDH13, among which *MdWRKY70L* was co-induced by drought and salt stress. Its overexpression in transgenic tobacco plants enhanced stress tolerance to both drought and salt. *GbWRKY1* is another WRKY transcription factor that plays a crucial role in the regulation of phosphate starvation, participating in the regulation of phosphate homeostasis, as well as the distribution and mobilization of phosphate. Overexpression of *GbWRKY1* in *Arabidopsis* can reduce phosphorus deficiency symptoms, accumulate high levels of total phosphorus, increase lateral root development. and enhance phosphatase activity (Xu et al., 2012). In *Liriodendron chinense*, the expression patterns of several WRKY genes (e.g., *LchiWRKY33*, *LchiWRKY25*, and *LchiWRKY18*) were analyzed, and found that they may play functional roles in regulating the response of trees to cold, heat, or drought stress (Wu et al., 2022). Additionally, WRKY genes can enhance plant Cd tolerance or maintain metal ion homeostasis by regulating downstream functional genes. For instance, *AtWRKY12* negatively regulates Cd tolerance in *A. thaliana* by directly binding to the W-box of the *GSH1* promoter and repressing the expression of genes related to phytochelatin synthesis (Han et al., 2019). On the other hand, *AtWRKY13* enhances plant Cd tolerance by directly upregulating the ABC transporter PDR8 to promote the production of D-cysteine desulfurization enzyme and hydrogen sulfide in *Arabidopsis* (Zhang et al., 2020).

*Brassica juncea* L. is an annual herbaceous plant widely cultivated in China. It exhibits a wide variety of species, most of which are characterized by high biomass, rapid growth, high tolerance, and other distinctive features, allowing for multiple cultivation each year (Chen L. et al., 2020). Previous studies have shown that *B. juncea* has the potential to tolerate and accumulate heavy metals and is a good candidate for phytoremediation in heavy metal contaminated soils (Du et al., 2020). Specifically, the maximum Cd accumulation potential of *B. juncea* shoots has been reported to be over 400 μg g^-1^, indicating its potential as a promising genotype for remediation of Cd-contaminated soils (Szczygłowska et al., 2011). Several genes related to heavy metal accumulation and tolerance have been identified in *B. juncea*. For instance, mustard *HMA2* and *HMA4* have been reported to play an important role in Zn detoxification and chelation at subcellular level (Meraklı and Memon, 2023). BjCET1 is a metal ion transporter protein that enhances the heavy metal tolerance in yeast cells when heterologous expressed, reducing the accumulation of Cd or zinc. Moreover, constitutive expression of *BjCET1* rescues the heavy metal tolerance capacity of transgenic tobacco plants (Han et al., 2022). Furthermore, chain-specific transcriptome sequencing and miRNA sequencing were employed to conduct genome-wide identification and characterization of miRNAs and their target genes in *B. juncea* leaves and roots exposed to Cd stress. The results revealed that bra-miR172b-3p is a potential cadmium-specific resistance suppressor that may be negatively regulated in *ATCCS* (copper chaperone for superoxide dismutase) under Cd stress. These findings contribute to deeper understanding of the regulatory network of Cd-responsive miRNAs in *B. juncea* (Liu et al., 2021).

In this study, we systematically identified and characterized a comprehensive repertoire of 291 WRKY transcription factor genes within the *B. juncea* genome. Subsequently, we conducted a thorough examination of their structural features, conserved motifs, cis-regulatory elements, and inferred their evolutionary relationships through phylogenetic analysis. Employing an integrated approach combining RNA sequencing (RNA-seq) and quantitative reverse transcription polymerase chain reaction (qRT-PCR), we carefully elucidated the expression patterns of these WRKY genes under Cd stress. Moreover, we explored the functional importance of key hub genes that orchestrate plant responses to Cd stress. The findings from this study provide a strong foundation for future research aimed at elucidating the functional roles of *BjuWRKY* genes in mediating plant resilience to Cd stress.

## 2 Materials and methods

### 2.1 Identification of WRKY genes in *Brassica juncea*

The *A. thaliana* (TAIR_V10. 1) and *B. juncea* genomic data related to this study were obtained from BRAD (http://brassicadb.cn) (Chen et al., 2022; Yang et al., 2021). All potential BjuWrky proteins were systematically identified and retrieved using the Hidden Markov Model (HMM) search with the hmmsearch software and default parameter settings. The strict criterion for inclusion was that each protein must contain the characteristic WRKY structural domain (accession number PF03106) (Mistry et al., 2013; Tang et al., 2021). Also, we used the Conserved Structural Domain Database (CDD; https://www.ncbi.nlm.nih.gov/Structure/cdd/wrpsb.cgi) and SMART (https://smart.embl-heidelberg.de/) to independently verify the presence and integrity of conserved WRKY structural domains in the identified sequences. Subsequently, molecular weights and theoretical isoelectric points (pIs) of the amino acid sequences were calculated using the ExPASy (https://web.expasy.org/). In addition, the PSORT (http://psort1.hgc.jp/form.html) was applied to infer the possible subcellular localization of these proteins. Finally, all identified sequences were systematically named according to their respective chromosomal locations to ensure clarity and consistency of nomenclature throughout the study.

### 2.2 Phylogenetic tree construction and conserved motifs identification

To elucidate the evolutionary relationships between *B. juncea* and *A. thaliana* WRKY transcription factors, a phylogenetic tree was meticulously constructed. Initially, multiple sequence alignment of the identified WRKY proteins was performed using ClustalW software with default parameter settings to ensure optimal alignment accuracy and consistency. This alignment served as the basis for subsequent phylogenetic inference. The Neighbor-Joining (NJ) method, implemented in the MEGA7.0 software package, was utilized for constructing the phylogenetic tree (Kumar et al., 2016). To enhance the robustness and reliability of the branching patterns, a bootstrap analysis with 1000 replicates was performed. The phylogenetic tree was visualized using iTOL (https://itol.embl.de/).

### 2.3 Gene structure, conserved motif, and promoter analysis

The structural information of the WRKY gene, including introns, exons, and untranslated regions, was downloaded from BRAD and analyzed using TBtools (Chen C. et al., 2020). The conserved motifs present within the full-length sequences of the identified WRKY proteins were systematically characterized using the online Multiple Em for Motif Elicitation (MEME) suite (http://meme-suite.org/), with the default program setting, where the maximum number of motif was 10 (Bailey et al., 2009).

To gain insights into the regulatory mechanisms governing the expression of *BjWRKY* genes, a 2000 base pair (bp) region immediately upstream of the translational start codon “ATG” was retrieved from the *B. juncea* genome database for each identified gene. These promoter sequences were subjected to analysis using the PlantCARE database. To facilitate the interpretation and visualization of these complex datasets, the TBtools software was employed.

### 2.4 Chromosomal location and duplication analysis

Chromosomal localization information for each *BjuWRKY* gene was obtained from BRAD, and gene localization visualization with TBtools was used to construct chromosomal localization maps for all *BjuWRKYs* (Chen C. et al., 2020). Gene duplication events of *BjuWRKY* genes were analyzed by WGDI (v0.6.1) software (Sun et al., 2022) and visualized by shinyCircos. Each of the duplicated *BjuWRKY* gene pairs was aligned using the ParaAT 2.0 with MUSCLE (v3.8.31) as multiple sequence aligner (Edgar, 2004; Zhang et al., 2012). The number of nonsynonymous (Ka) and synonymous (Ks) substitutions per duplicate *BjuWRKY* gene was calculated using KaKs_Calculator 3.0 (Zhang, 2022).

### 2.5 Plant materials and Cd stress treatments

The leaf mustard “137”, selected and cultivated by the Zhejiang Academy of Agricultural Sciences, was utilized as the test material. The seeds were germinated on wet filter paper at 26°C. The germinated seedlings were then cultured in a growth chamber at 14 h/10 h (day/night) cycle for 7 d at 26°C. Subsequently, the seedlings were transplanted to one-half Hogan’s nutrient solution for hydroponic cultivation, with the culture solution being changed every 5 days. 21 days, uniformly sized seedlings were selected for 100 μmol Cd stress treatment. Roots, stems, and leaves were sampled at 0 d, 1 d, and 4 d after Cd stress treatment, respectively. All samples were collected in three biological replicates.

### 2.6 *BjuWRKY* expression profiles in response to Cd stress and qRT-PCR expression analysis

Total RNA was isolated and converted into cDNA using TRIzol Reagent (Invitrogen, Waltham, MA, USA) and PrimeScript™ RT Master Mix (TaKaRa), respectively. The transcript levels were quantified using SYBR Green Premix Ex Taq II (TaKaRa) on a 7300 Real-Time PCR (Applied Biosystems, Carlsbad, CA, USA). Three independent experiments were performed, with three biological replicates per treatment.

To normalize gene expression levels, the 2^-ΔΔCt^ method was calculated. The *TIP41*(tonoplastic intrinsic protein 41) gene was used as the reference (Zheng et al., 2022). Primers used are listed in **Supplementary Table S1**.

The transcriptome of roots, stems and leaves of mustard at three time points under Cd stress was analyzed using RNA-seq. In this study, twenty-seven RNA libraries were generated, including three control libraries and twenty-four treatment libraries, to investigate the effect of treatment on gene expression. Raw reads were analyzed using FastQC (https://www.bioinformatics.babraham.ac.uk/projects/fastqc/) prior to assembly. Cutadapt software was used to remove low quality reads, including those with sequencing adapters, sequencing primers and nucleotides with mass fractions below 20. All downstream analyses were based on high quality clean data. The raw sequence data was submitted to the NCBI database under the registration number PRJNA1133808. The expression values were calculated by log2 (FPKM) and normalized via Z-score normalization. An expression profile heatmap was generated using the heatmap package in R (4.0.2).

### 2.7 Gene ontology annotation analysis and hub genes identified

For GO annotation analysis of all identified WRKY protein sequences, Blast2GO v3.0.11 (https://www.blast2go.com) and OmicsBox software were utilized (Conesa and Gotz, 2008). Based on the *Arabidopsis* correlation model, a functional protein association network was constructed in the STRING program (https://string-db.org/) with a confidence parameter of 0.4 and an interaction number of 5. The network was further visualized and analyzed using Cytoscape version 3.10.2.0.

The Cytoscape plugin cytoHubba offers various topological algorithms for ranking nodes base on their correlation to the network topology. We chose to use the Maximum Cluster Centrality (MCC) algorithm, which is based on identifying interconnections between genes and the maximum number of cliques to which a node may be affiliated (Chin et al., 2014; Liu et al., 2022). The Cytohubba plugin in Cytoscape was used to determine hub genes in the target module.

## 3 Results

### 3.1 Identification and characterization of WRKY transcription factors in *Brassica juncea*

The BjuWRKY proteins were searched and characterized from the BRAD database using Hidden Markov Models (HMM), and the identified candidate sequences were examined using CDD, Pfam, and SMART to confirm the WRKY structural domains (e-value < 1e^-5^). A total of 291 WRKY family members were finally identified from the *B. juncea* genome (**Supplementary Table S2**). The BjuWRKY family genes were sequentially named from *BjuWRKY1*-*BjuWRKY291* based on their location on the chromosome. The length of these genes ranged from 348bp (*BjWRKY31*)-3285bp (*BjWRKY157*), and the corresponding molecular weights of the proteins were between 13323.75 and 121396.56 Da (Dalton), and the isoelectric points (pI) ranged from 4.82-9.92. The subcellular localization of BjuWRKY proteins was predicted using the PSORT tool. Most of the BjuWRKY proteins localized in the nucleus. A few proteins, such as BjuWRKY91, BjuWRKY164, BjuWRKY201, and BjuWRKY283 localized in the chloroplasts. BjuWRKY101, BjuWRKY174 and BjuWRKY196 are localized in the cell membrane, while BjuWRKY167 was localized in both the nucleus and chloroplasts. Except for five proteins (BjuWRKY101, BjuWRKY167, BjuWRKY196, BjuWRKY201, and BjuWRKY234), the other BjuWRKY proteins had no transmembrane structures, indicating that they are not membrane proteins (**Supplementary Table S3**).

### 3.2 Phylogenetic analysis of *BjuWRKY* genes

**Figure 1:**
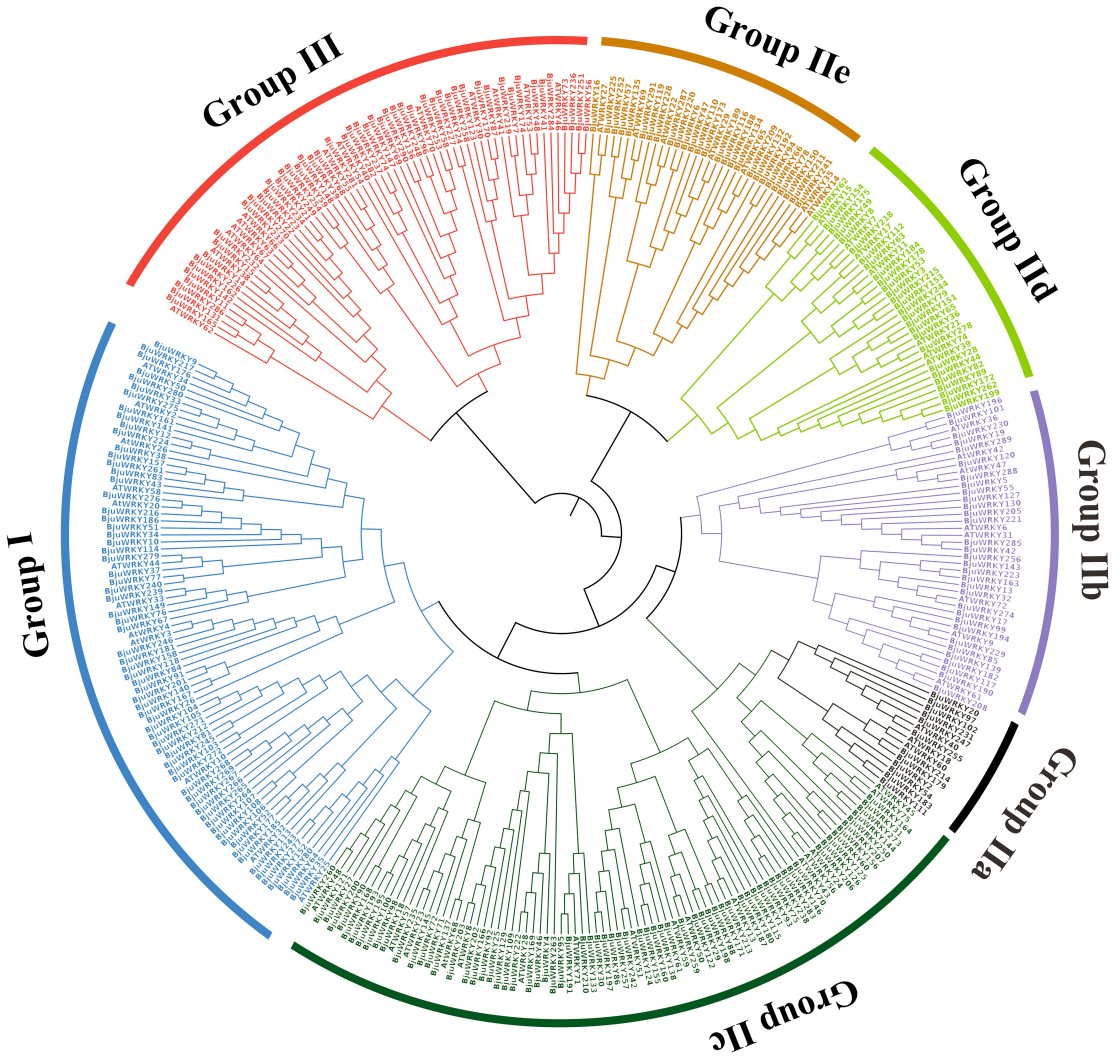
Phylogenetic relationships among the WRKY genes from *B. juncea* and *Arabidopsis*. The phylogenetic tree was constructed on the basis of the alignment of *B. juncea* and *Arabidopsis* WRKY proteins according to the maximum-likelihood method, with 1,000 bootstrap replicates.

To further explore the classification of subgroups of *BjuWRKY* genes and to elucidate the evolutionary relationship between *AtWRKY* and *BjuWRKY* genes, we constructed a phylogenetic tree using the maximum likelihood method (**Figure 1**). The phylogenetic tree showed that 291 *BjuWRKY*s were categorized into three subgroups: group I, II, and III. The group II contained the most WRKYs with 65.98%, while group I and III contained 47 and 52 WRKYs, respectively. Groups II-a, -b, and -c were clustered together with group I, whereas groups II-d and -e were more closely related to group III. In addition, WRKY proteins in group II could be further divided into five subgroups (IIa, IIb, IIc, IId, and IIe) containing 12, 32, 94, 27, and 27 members, respectively (as shown in **Supplementary Table S3**). The classification results for these members are similar to those obtained for *Arabidopsis* Within the three groups, the abundance of WRKY proteins varies between Arabidopsis and mustard, potentially attributable to processes of gene expansion or loss. It is evident that in subgroup IIa, both branches contain one or two AtWRKY proteins, and the number of BjuWRKY proteins in each branch of these branches increases to six, indicating the potential expansion of the BjuWRKY family in this subgroup.

### 3.3 Conserved motifs and gene structures of WRKY family in *B. juncea*

**Figure 2:**
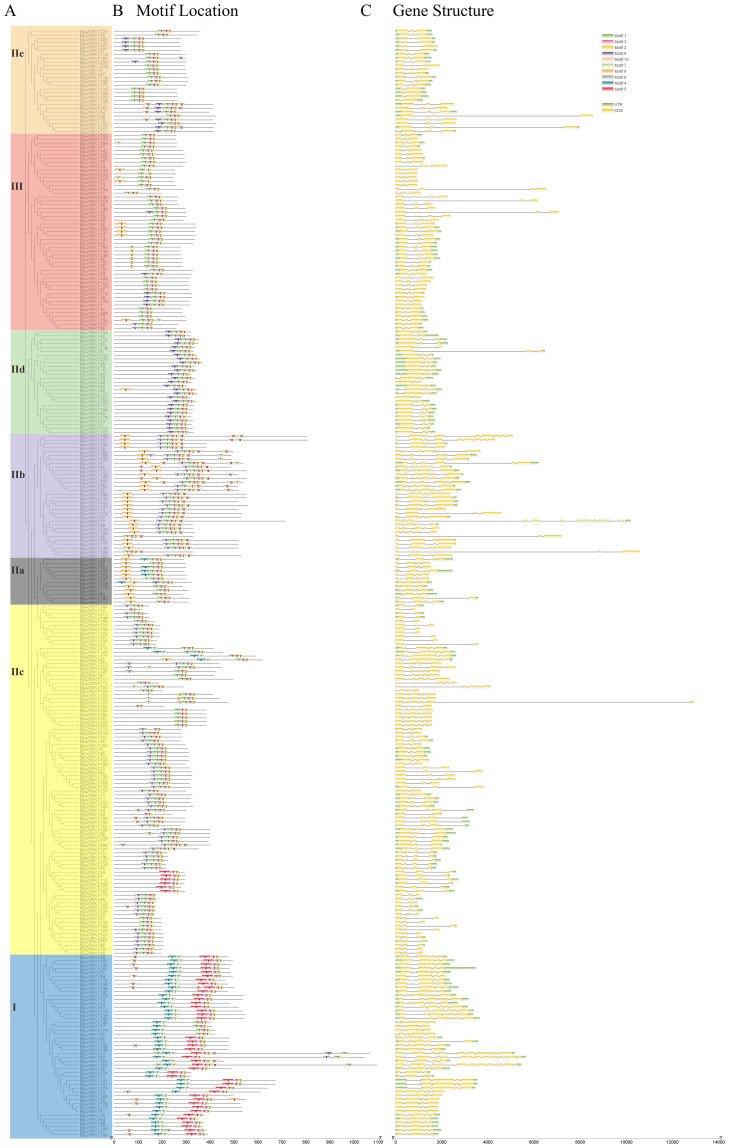
Phylogenetic relationships, motif compositions, and gene structures of *BjuWRKY* genes in *B. juncea*. **(A)** Phylogenetic analysis of *B. juncea* WRKY family members. **(B)** All conserved motifs in the WRKY proteins were identified using the MEME program. Different motifs are highlighted with different colored boxes (numbered 1–10). **(C)** Gene structures. Exons and 5’/3’ untranslated regions are indicated by green and yellow bars, respectively, whereas gray lines represent introns.

To further elucidate the structural features of conservation and diversification of BjuWRKY proteins, all BjuWRKY proteins were subjected to motif analysis using MEME. Details regarding the 10 putative motifs are provided in **Supplementary Table S4**. A total of 10 different motifs were identified, and the conserved motifs contained 20-50 amino acids. The number of conserved motifs for each BjuWRKY protein was 3 ~ 7 (**Figure 2B**). The motif2 and motif3 were present in all of the BjuWRKYs.

To further understand the gene structure of *BjuWRKY*s, we analyzed the introns and exons of *BjuWRKY* genes using the annotation information. The number of introns in the family of *BjuWRKY*s ranged from 1 to 15. Seventeen genes contained only one intron, and 146 (0.18%) and 59 (20.28%) genes contained two and three introns, respectively. Four or more introns were detected in 69 genes (**Figure 2C**). Combined with the gene structure and conserved protein motif analysis of BjuWRKY members, we found that members of the same subfamily have similar gene structures and conserved motif distributions among themselves, whereas different subfamilies have greater variations, suggesting that there is conservatism and similar functions within the same subfamily.

### 3.4 Analysis of cis-elements in the promoters of the *BjuWRKY* genes

**Figure 3:**
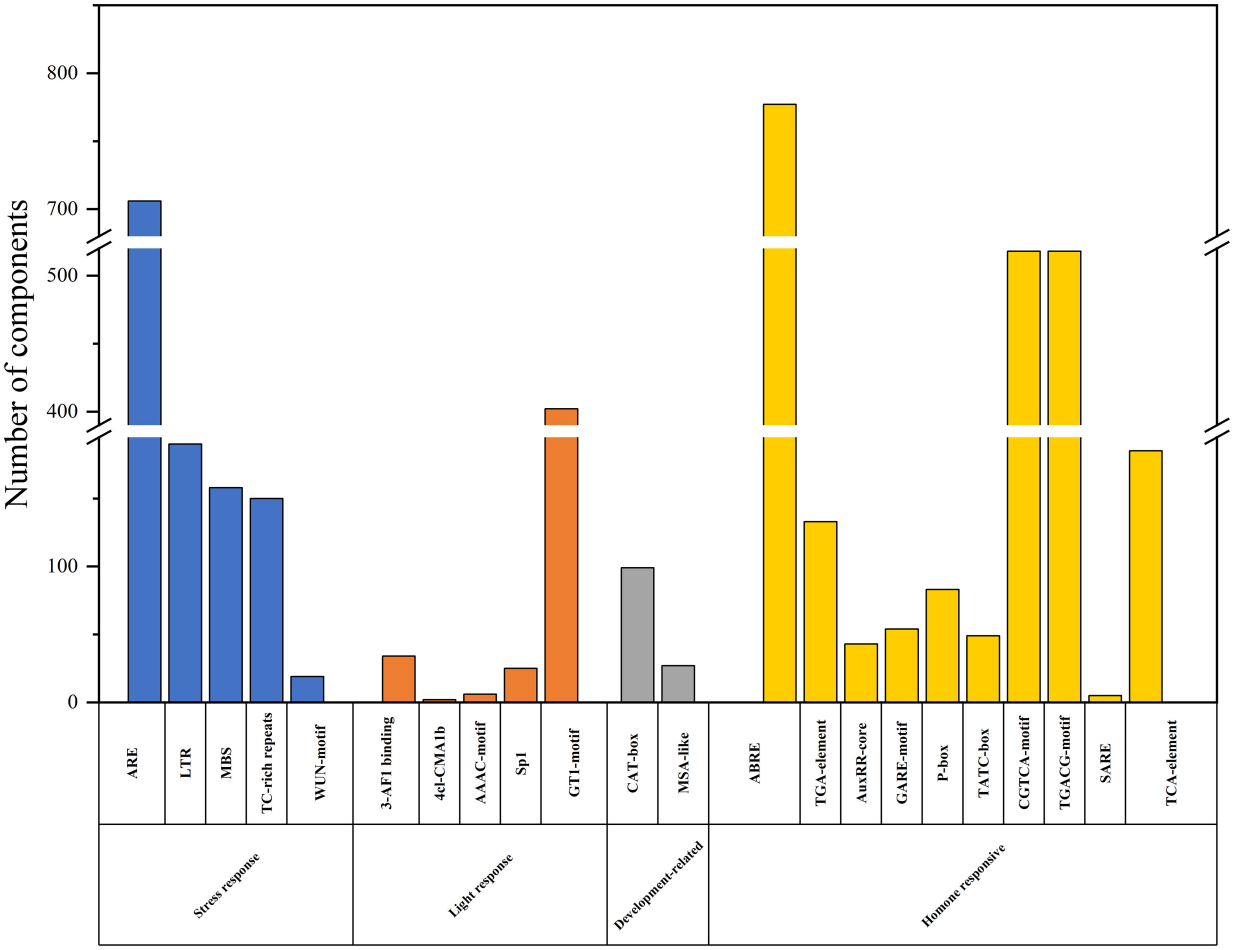
Cis-regulatory elements analysis in the promoter region of *BjuWRKY* genes. Cis-acting elements are divided into four categories based on functional annotation: stress response, light response, development-related, hormone responsive cis-acting elements.

To gain a deeper understanding of the function and regulatory mechanism of *BjuWRKY* genes, and to elucidate the role of cis-acting elements in *BjuWRKY*. The upstream 2000 bp sequence of each *BjuWRKY* gene was selected as the promoter sequence. The cis-acting elements in these promoter sequences were analyzed using PlantCARE and visualized using TBtools (**Supplementary Figure S1**). The identified cis-acting elements were categorized into four classes: stress-responsive, light-responsive, plant development-related, and phytohormone-responsive. Plant stress-responsive cis-acting elements included low-temperature-responsive (LTR), defense- and stress-responsive (TC-rich), drought-inducible (MBS), and trauma-responsive (WUN-motif); and phytohormone-responsive cis-acting elements, including abscisic acid-responsive (ABRE), growth hormone-responsive (AuxRR-core and TGAelement), gibberellin-responsive elements (GARE-motif, P-box, and TATC-box), MeJA-responsive (CGTCA-motif and TGACGmotif), and salicylic acid-responsive (TCA-element and SARE) were widely present in the promoter region. Among these elements, ABA- and MeJA-related elements (ABRE, CGTCA-motif, and TGACG-motif) accounted for the largest portion, whereas SARE elements were found only in the promoter regions of *BjuWRKY38*, *BjuWRKY46*, *BjuWRKY47*, and *BjuWRKY169*, and *BjuWRKY280*, suggesting that these five genes may play a role in the SA signaling pathway. Other elements related to development and light response, such as GT1-motif and CAT-box were also found in the promoters of several WRKY genes (**Figure 3**).

### 3.5 Chromosomal locations and collinearity analysis of *BjuWRKY* genes

**Figure 4:**
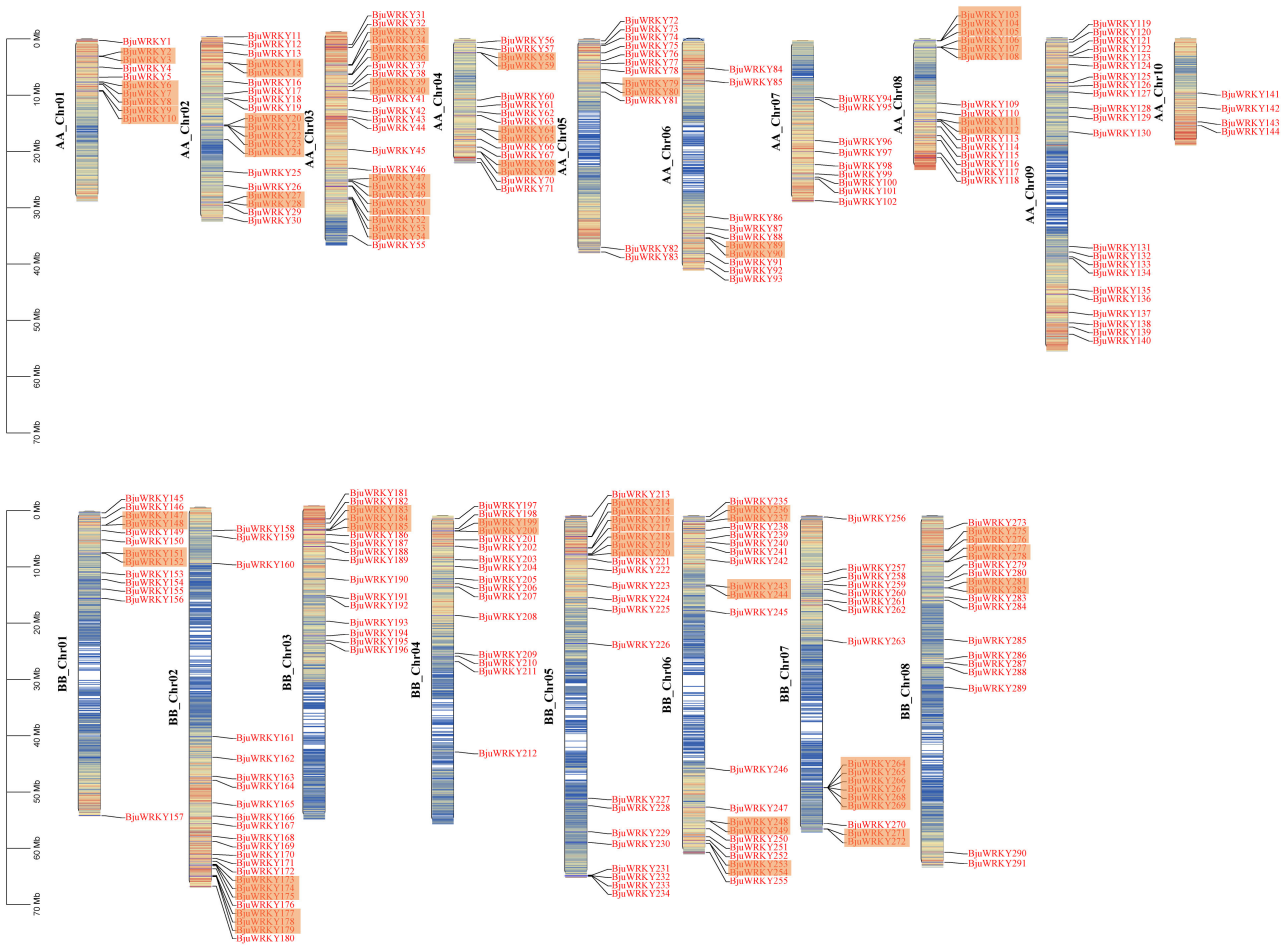
Distribution of the 291 *BjuWRKY* genes in the *B. juncea* genome. The colored columns represent chromosomes with the gene names shown on the right. Chromosome numbers are listed on the left, while chromosome sizes are indicated on the left side of the figure. The length of each chromosome on the left was estimated in mega base (Mb). The orange shadow represents tandem repeat genes.

Based on the annotation information of the *B. juncea* genome, we mapped the distribution of genes on chromosomes. The **Figure 4** shows that 291 genes were unevenly distributed on 18 chromosomes, with the number of genes distributed on chromosomes of subgenomes A and B was almost the same. The highest number of genes was found in ChrA03 (25), followed by ChrB02, ChrA09 and ChrB05 with 23, 22 and 22 genes, respectively. The lowest number of individual genes were ChrA10, ChrA07 and ChrA01, ChrA06.

**Figure 5:**
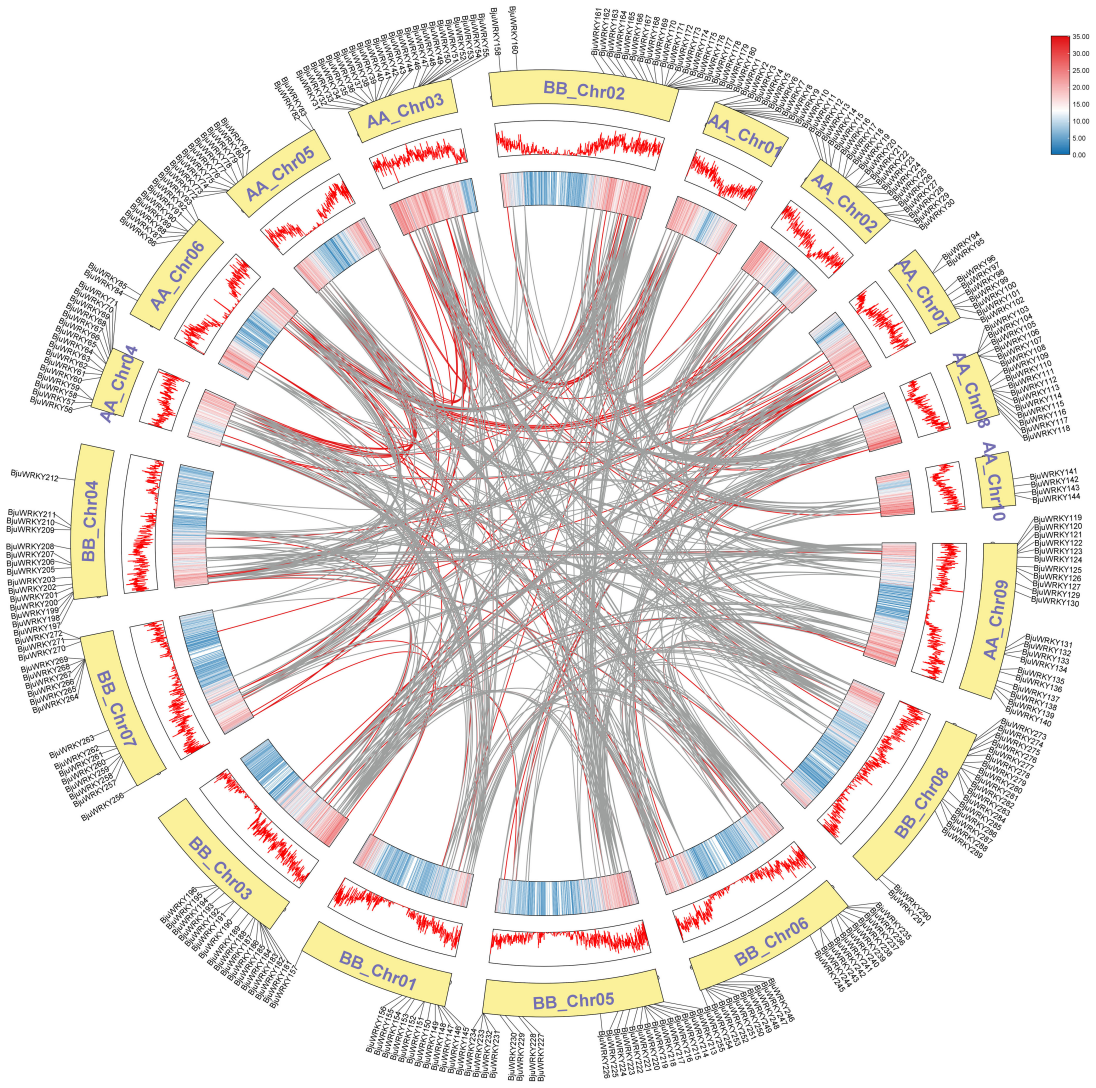
Synteny analysis of WRKY genes. Chromosomes were represented by yellow boxes with the gene names surrounding the boxes. Box with red line and colored box represents gene density. Segmental duplication gene pairs of *B. juncea* WRKY genes are linked by red lines. Gray lines indicate syntenic blocks in the *B. juncea* genome.

We also analyzed the replication events of the *BjuWRKY* gene. Gene duplication plays an important role in the amplification of gene families and their subsequent evolution. When two or more genes are located within a 200 kb chromosomal region, they are considered to be the result of tandem duplication events (Holub, 2001). In *BjuWRKY*, about 31.62% (92 out of 291) of the genes were found to have originated from tandem duplication events. The set of 39 tandemly duplicated *BjuWRKY*s contained 2-6 *BjuWRKY* genes. Specifically, the set of 29 tandemly duplicated *BjuWRKY*s contained 2 members, the set of 8 tandemly duplicated *BjuWRKY*s contained 3 members, and 4 and 6 members were involved in the set of 1 tandemly duplicated *BjuWRKY* (**Supplementary Table S5**), respectively. In addition to tandem duplication events, we observed that *BjuWRKY* genes are involved in segmental duplication, which indicates gene transfer and a change in the chromosome set (**Figure 5**). In addition, only about 2% of the *BjuWRKY* genes had ka/ks ratios exceeding 0.5 (**Supplementary Figure S2**), indicating that members of the *BjuWRKY* family are mainly under purifying selection.

### 3.6 *BjuWRKY* expression profiles under Cd stress conditions

**Figure 6:**
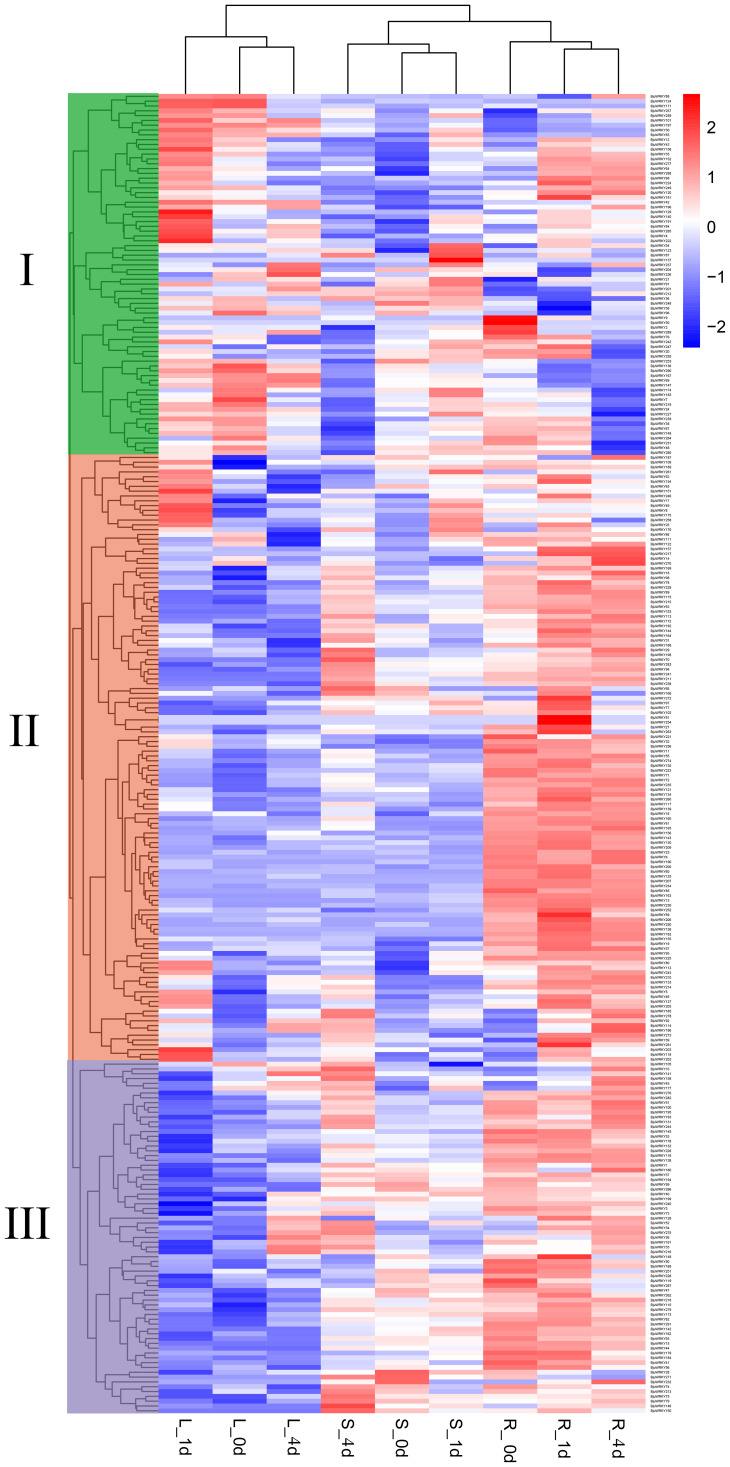
Expression analysis of *BjuWRKY* genes in several organs. The 0d, 1d, and 4d tags indicate the time-points when the samples were harvested. In the expression bar, the red color shows high, and the blue color shows low, expression levels. The expression heat map was created using FPKM values.

To investigate the expression pattern of *BjuWRKY* genes in mustard under Cd stress, we obtained RNA-seq data from different organs of mustard. The dynamic expression patterns of 291 genes after Cd treatment were analyzed (**Supplementary Table S6**). The roots, stems and leaves were collected at 0 d, 1 d and 4 d of Cd treatment. The differential expression patterns of all genes were analyzed and grouped into 3 clusters (I, II, III) (**Figure 6**). Cluster I contained 75 *BjuWRKY* genes, cluster II 73 *BjuWRKY* genes, and cluster III 143 *BjuWRKY* genes. In roots, genes in cluster I showed different degrees of up-regulated expression at various time points after Cd treatment; in stems, most of them were up-regulated after 1 d of treatment and down-regulated for 4 d of treatment. In addition, most of the genes showed up-regulated expression in leaves, especially at 1 d of treatment. In contrast, most members of cluster II had the highest expression in roots, especially in leaves treated with Cd for 1 d. Only 13 genes did not show up-regulated expression. In cluster III, most of the genes in roots and stems showed an up-regulated trend.

### 3.7 Functional annotation and interaction network analysis of BjuWRKY proteins

**Figure 7:**
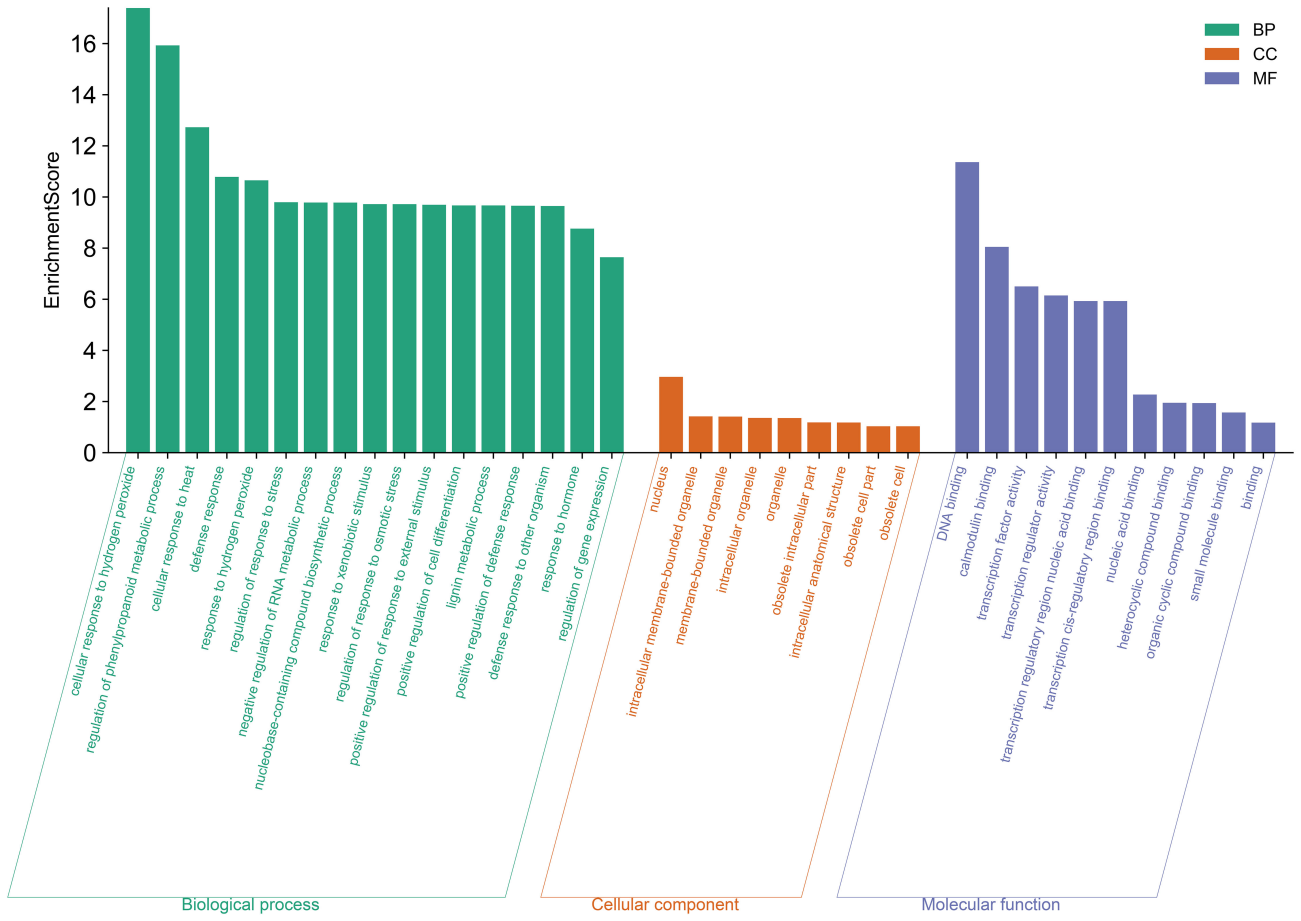
GO and functional annotation of BjuWRKY.

**Figure 8:**
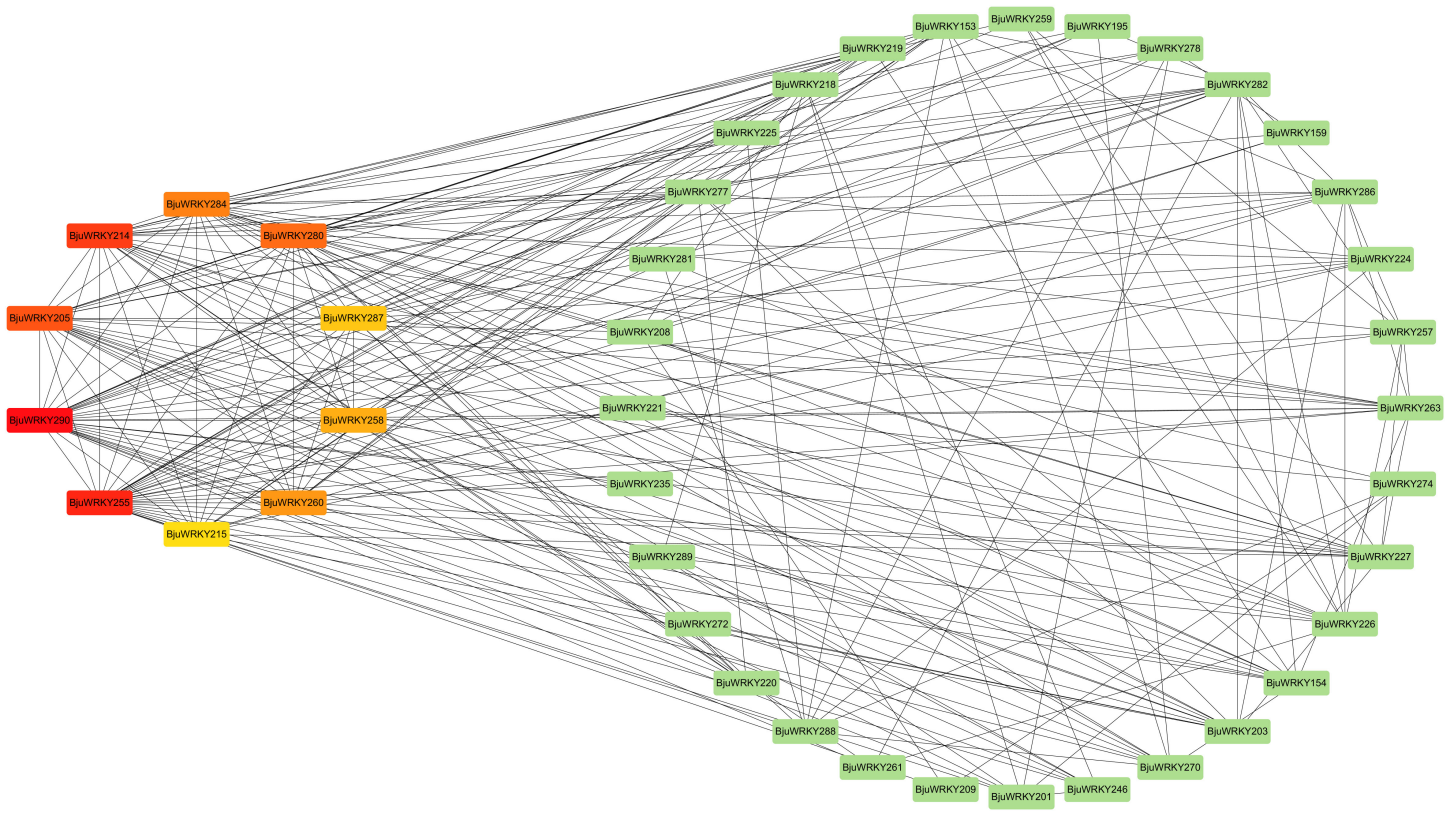
Interaction network of BjuWRKY factors in *B. juncea* according to orthologs in *Arabidopsis*.

To better characterize the function of *BjuWRKY* genes, we performed GO annotation and enrichment analyses based on the categories of biological process (BP), molecular function (MF), and cellular component (CC); these terms contribute to our comprehension of the gene’s function at the molecular level (**Figure 7**). The GO-BP results predicted a large number (17) of significantly enriched terms. The most common and useful terms are cellular response to hydrogen peroxide (GO:0070301), regulation of phenylpropanoid metabolic process (GO: 2000762), cellular response to heat (GO: 0034605), defense response (GO: 0006952), response to hydrogen peroxide cellular response to stimuli (GO: 0042542), etc. GO-CC enrichment analyses revealed 9 high enrichments for nucleus (GO: 0005634), intracellular membrane-bounded organelle (GO: 0043231), membrane-bounded organelle (GO: 0043227), intracellular organelle (GO: 0043229), organelle (GO: 0043226). The GO-MF enrichment detected 11 highly enriched terms, which included DNA binding (GO: 0003677), calmodulin binding (GO: 00905516), transcription factor activity (GO:0003700), transcription regulator activity (GO:0140110), and transcription regulatory region nucleic acid binding (GO: 0001067), etc. Overall, GO enrichment analysis confirmed the functional role of the *BjuWRKY* genes in cellular, molecular, and biological processes related to antioxidant enzymes, subcellular localization, and stress response. The Cytoscape plugin cytoHubba (Maximum Cluster Centrality (MCC) algorithm) was applied to identify 10 hub genes (**Figure 8**). Annotation of the 10 hub genes showed that they are mainly involved in stress response and specific binding (**Supplementary Table S7**).

### 3.8 Analysis of the expression pattern of key WRKY genes in different tissues under Cd stress

**Figure 9:**
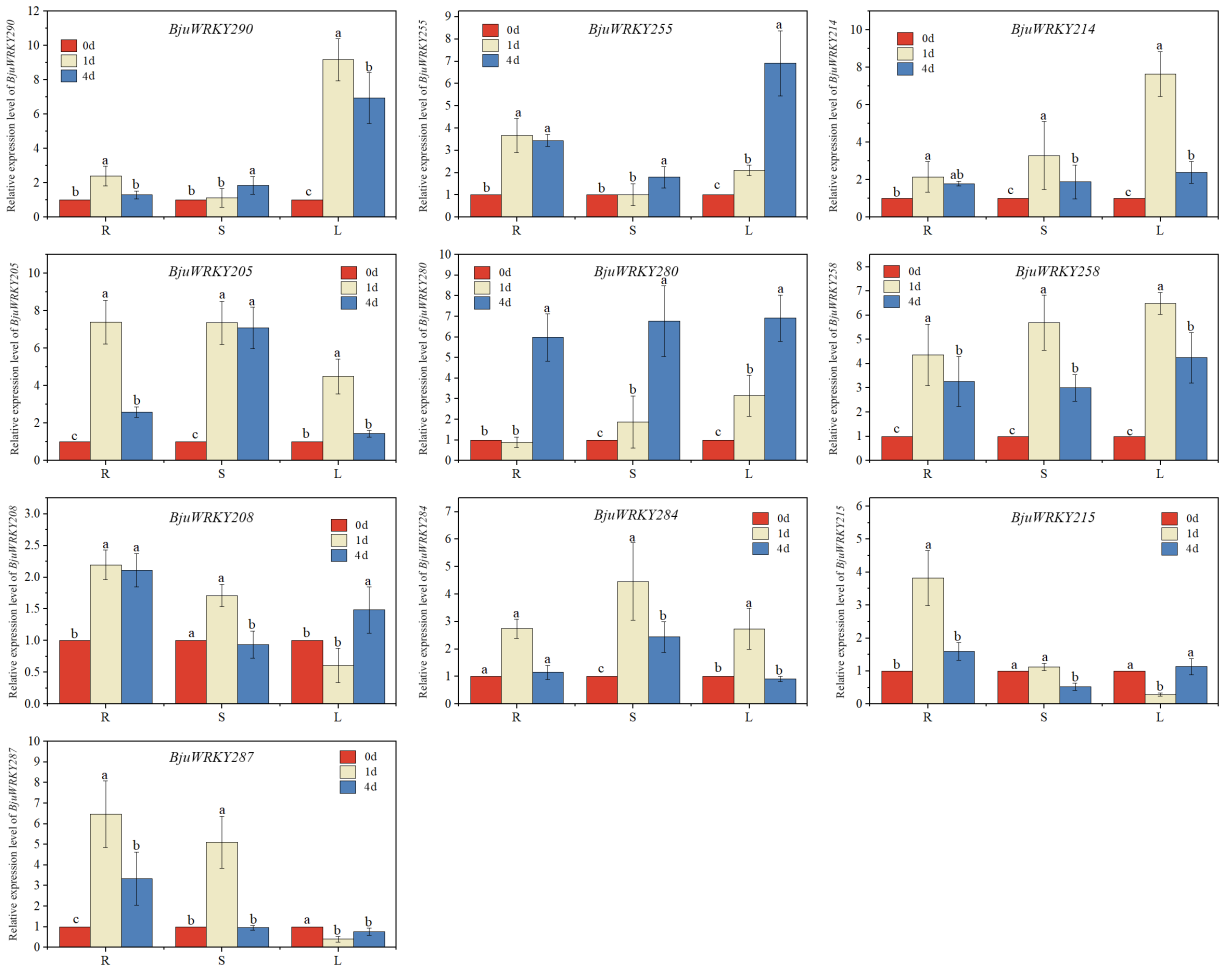
qRT-PCR verification of the Cd stress response of 10 *BjuWRKY*s. Expression analysis of *BjuWRKY*s under Cd stress conditions, determined by qRT-PCR. (n=3, *p* < 0.05). Different letters above bars indicate significant differences.

The hub genes in the protein-protein interaction network were selected for expression level validation (**Figure 9**). The results revealed that most of the genes were induced to be up-regulated for expression by Cd stress. Except for *BjuWRKY208*, the remaining *BjuWRKY* key genes were significantly up-regulated in roots at 1d and 4 d of Cd stress. Only the *BjuWRKY280* gene did not show a trend of increased and then decreased expression in roots after Cd stress. In stems, only *BjuWRKY290*, *BjuWRKY280*, and *BjuWRKY255* showed a gradual increase in expression after Cd stress, while the remaining genes displayed an increase followed by a decrease in expression trend. It is noteworthy that the *BjuWRKY215* gene was not induced to be expressed in stems treated with Cd for 1 d and 4 d. Notably, *BjuWRKY287* and *BjuWRKY215* were not induced to be expressed by Cd in leaves. In addition, *BjuWRKY205* and *BjuWRKY284* were significantly up-regulated for expression by Cd at 1 d and were not induced at 4 d. The opposite trend was observed for *BjuWRKY280*, whose expression was not induced by Cd treatment at 1 d and was significantly up-regulated by Cd treatment at 4 d.

Furthermore, we performed a correlation analysis between the RNA-seq and qRT-PCR results for ten selected genes. A correlation heatmap was established using the Sperman correlation method. The expression patterns of these genes exhibit a fundamental consistency with the transcriptome results, demonstrating a high correlation and thus indicating the reliability of the transcriptome data (**Supplementary Figure S3**).

## 4 Discussion

The *B. juncea* known for its widespread cultivation as a vegetable and oilseed worldwide, exhibits rapid growth and high biomass production (Kang et al., 2021; Singh et al., 2021). Its ability to tolerate and accumulate Cd confers a significant advantage in phytoremediation applications, allowing it to remediate soil while maintaining economic benefits (D’Alessandro et al., 2013; Li L. et al., 2023). Although multiple gene families related to *B. juncea* development and abiotic stresses have been reported (Khuman et al., 2024; Li et al., 2020; Verma et al., 2023), relatively few studies have been focused on heavy metal tolerance or sensitivity genes in *B. juncea*. The mechanisms of transport, uptake, and tolerance to Cd in *B. juncea* remain incompletely understood. Given that transcription factors of the *WRKY* family play pivotal roles in plant defense responses and secondary metabolism, studying the *BjuWRKY* family is crucial for elucidating the response mechanism of *B. juncea* to environmental factors, particularly heavy metal stress.

Transcription factors are essential in controlling plant development and stress responses. The WRKY transcription factors are not only in higher plants but also in other eukaryotes (Zhang and Wang, 2005). The publication of the genomes for *BjuWRKY* and its parents has enabled the identification of gene families associated with heavy metal response at the genome-wide level (Kang et al., 2021). In this study, we conducted a genome-wide analysis of the *B. juncea* WRKY transcription factor family and explored its potential functions under Cd stress. In the intricate plant evolution, gene expansion events emerge as pivotal forces driving the number of gene families, thereby contributing to the diversity and complexity of plant genomes (Xie et al., 2022). under this underground, we identified 291 WRKY genes in the *B. juncea* genome, significantly more than in *A. thaliana* (74) and *Oryza sativa* (109) (Eulgem and Somssich, 2007; Sahebi et al., 2018). The large number of genes may be attributed to duplication events during genome evolution. Related studies suggest that tandem and segmental duplication events are the primary drivers of WRKY evolution in plants, closely related to the expansion of gene functions and environmental adaptation (Chang and Duda, 2012). According to our statistics, 74 pairs of tandem duplicates and 274 pairs of segmental duplicates are present in the *B. juncea* gene family, with a Ka/Ks ratio less than 0.5, indicating that the genes have experienced repetitive events and strong purifying selection pressures during the evolutionary process. Similar to typical WRKY family proteins in other species, the 291 WRKY genes were classified into groups I, IIa, IIb, IIc, IId, IIe, and III (Yang et al., 2023). The amino acid sequences of the WRKY proteins are highly conserved (**Supplementary Figures S1**–**S3**), with proteins in the same group sharing the same protein motifs. For instance, motifs 2 and 3 are present in all *BjuWRKYs*, and motifs 4,5,7 occur together in group I.

The expression pattern of WRKY gene in different tissues provides valuable insights into their functionality. Using transcriptome data, our analysis revealed that the expression of WRKY family genes varied in different tissues (**Figure 6**). The expression patterns of WRKY genes in various tissues and under abiotic stresses have been reported for several species. For instance, EjWRKY17 has been shown to enhance drought tolerance in transgenic *A. thaliana* (Wang et al., 2021). Similarly, overexpression of *AtWRKY30* significantly elevated resistance to salt stress in *A. thaliana* (Scarpeci et al., 2013). Furthermore, overexpression of *GmWRKY142* in *A*. *thaliana* and soybean hairy roots leads to decrease Cd uptake and positively regulate Cd tolerance (Cai et al., 2020).

The promoter region of the *BjuWRKY* gene contains various hormone and stress response elements, with some elements such as ARE, GTI-motif, ABRE, CGTCA-motif, and TGACG-motif exceeding 400 occurrences. This suggests that these genes may play important roles in response to stress, hormones, light, and developmental processes. In addition, GO enrichment analysis demonstrated that the molecular functions of *BjuWRKY* are primarily involved in stress- and defense-related processes. The highest number of genes was found in cellular component processes, which is consistent with our results on the subcellular localization of BjuWRKY proteins. Based on the characterization of transcription factors, BjuWRKY proteins were significantly enriched in DNA binding, calmodulin binding, and transcription activity functions during molecular function processes.

Using String and Cytohubba plugin, we identified 10 hub genes, all of which were functionally annotated. In addition, WRKY TFs recognize and bind to the TTGAC(C/T) W-box in the promoter regions of target genes (Takeda, 2021). WRKY TFs can bind not only upstream promoters but also to upstream promoters of other genes regulated by WRKY TFs (Brand et al., 2013; Yang et al., 2023). Thus, it is speculated that in this network, the five hub genes annotated as “Interacts specifically with the W box”. It is speculated that in this network, they may regulate plant tolerance more by working together with target genes.

## 5 Conclusion

In this study, a comprehensive analysis of the WRKY gene family in mustard (*Brassica juncea*) was conducted, identifying a total of 291 WRKY family genes and elucidating their evolutionary relationships. These genes were systematically classified into three main groups, with group II further subdivided into five distinct subgroups based on their phylogenetic affinities. Each group member exhibited a conserved gene structure and motif composition, underscoring their evolutionary relatedness. A thorough examination of the basic features, gene structures, conserved motifs, and cis-regulatory elements of these *BjuWRKY* genes was performed, offering a foundational framework for understanding the *BjuWRKY* gene family. To investigate the expression patterns of *BjuWRKY* genes, RNA-seq and RT-qPCR analyses were employed. The results revealed that 10 selected *BjuWRKY* genes displayed tissue-specific expression profiles and were significantly influenced by Cd stress. These findings suggest that these genes may play crucial roles in modulating mustard’s response to Cd stress. To sum up, the study provides a solid foundation for further functional characterization of *BjuWRKY* genes implicated in stress resistance mechanisms in B. juncea, thereby advancing our understanding of the molecular basis of stress tolerance in this important crop species.

## Data Availability

The data presented in the study are deposited in the National Center for Biotechnology Information Sequence Read Archive repository, accession number PRJNA1133808. The names of the repository/repositories and accession number(s) can be found in the article/**Supplementary Material**.
