# Supplementary material for: Genome-wide analysis of WRKY gene family and the dynamic responses of key WRKY genes involved in cadmium stress in Brassica juncea
Source: Front Plant Sci. 2024 Oct 10;15:1465905. doi: 10.3389/fpls.2024.1465905 (PMC11499187; doi:10.3389/fpls.2024.1465905)
Supplement: Supplementary file 8 [file DataSheet1.pdf]

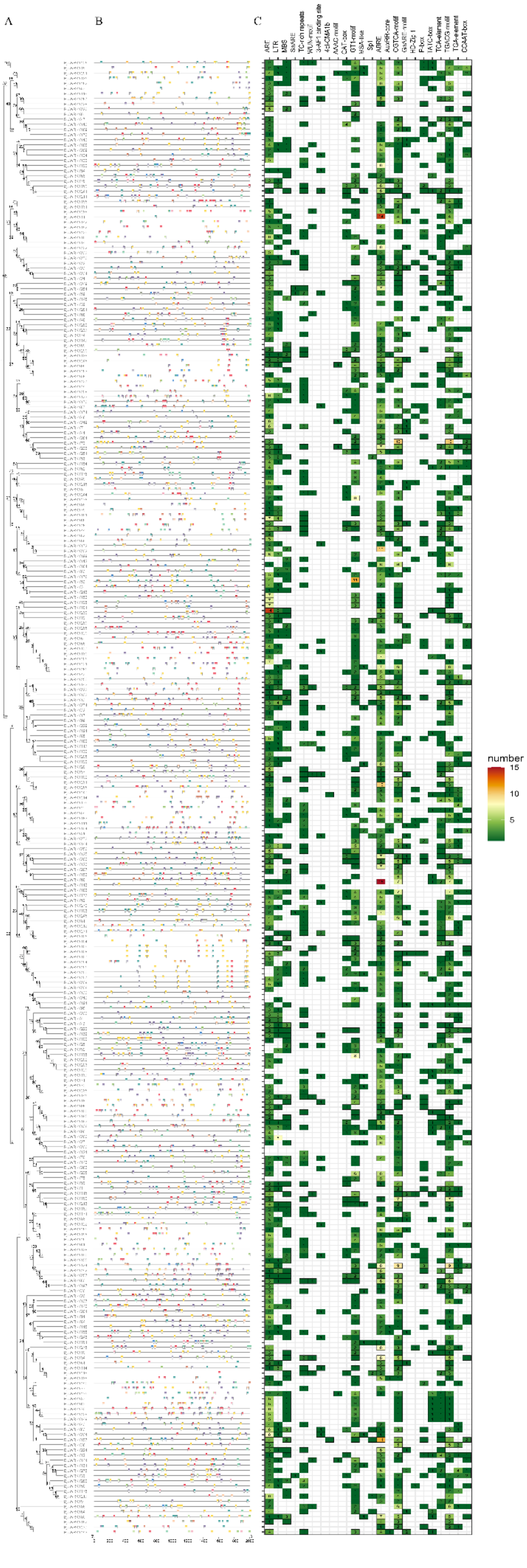
A
B
C
ARE
LTR
MBS
SaARE
TC-rich repeats
WUN-motif
3-AF1 binding site
4cl-CMA1b
MAAC-motif
CAT-box
GT1-motif
MSA-like
Sp1
ABRE
AuxRR-core
CGTCA-motif
GARE-motif
HD-Zip 1
P-box
TATC-box
TCA-element
TGACG-motif
TGA-element
CCAAT-box
number
15
10
5

Fig.S1 Analysis of cis-acting elements in the *BjuWRKY* promoter region. (A) Phylogenetic analysis of *BjuWRKY* genes. (B) Distribution of related cis-acting elements in *BjuWRKY* promoters. (C) The number of each cis-acting element in the promoter region (2 kb upstream of the translation start site) of *BjuWRKY* genes.

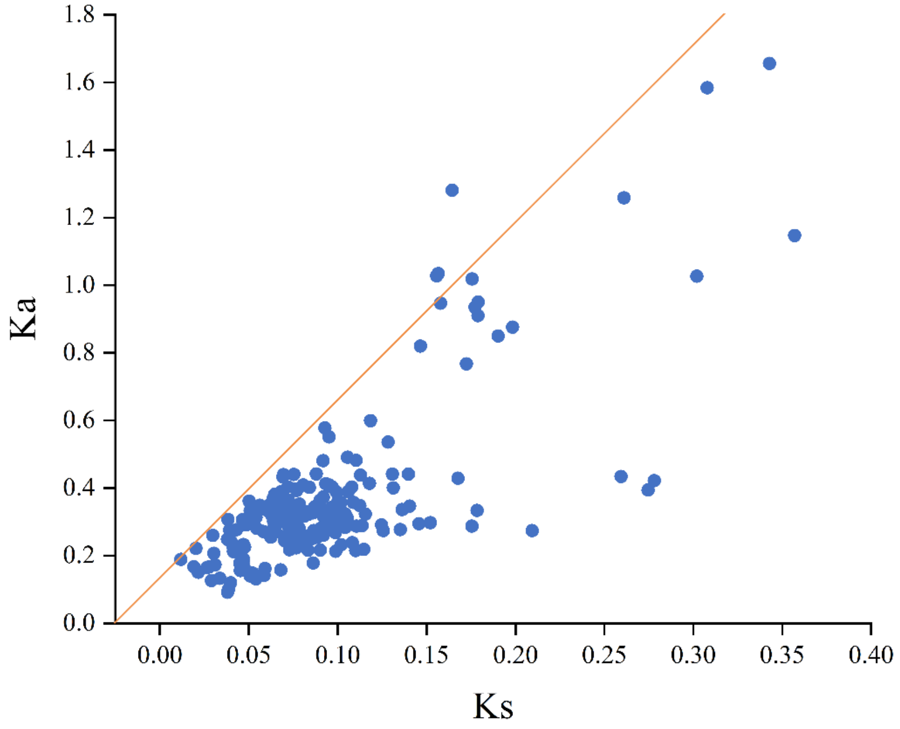

Fig.S2 Distribution of Ka and Ks from *B. juncea* gene pairs.

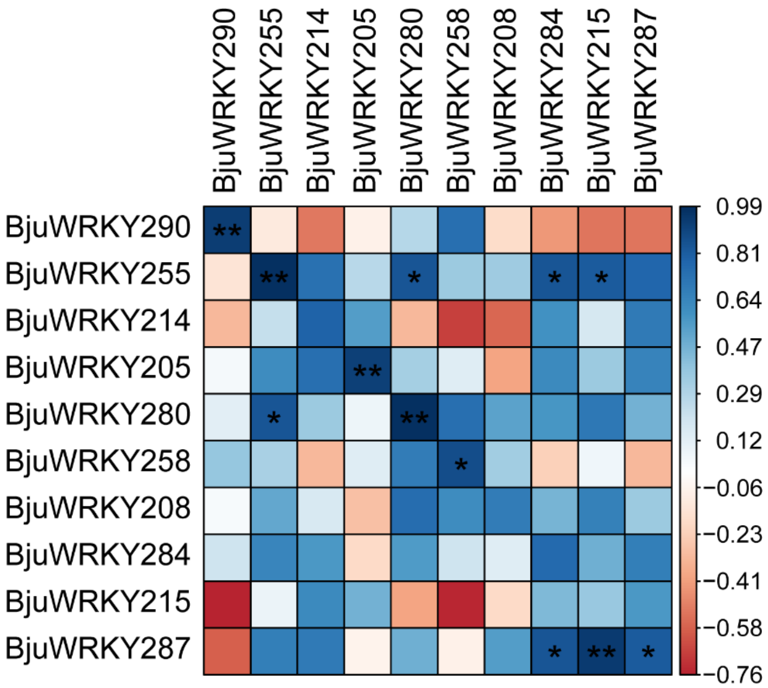

Fig.S3 The Sperman analysis between the RNA-seq and qRT-PCR results for ten hub genes. The colors of the box represent the degree of correlation; blue color represents the highest degree of correlation and red color represents the lowest degree of correlation. * $p < 0.05$, ** $p < 0.01$.
